# Heart Failure With Mildly Reduced Ejection Fraction: A Call for a Precision Medicine Approach

**DOI:** 10.1016/j.jacadv.2025.102476

**Published:** 2026-01-28

**Authors:** Lingling Wu, Affan Rizwan, Mario Rodriguez, Karim El Hachem, Hafeez Ul Hassan Virk, Muzamil Khawaja, Markus Strauss, Chayakrit Krittanawong

**Affiliations:** a Cardiovascular Division, University of Alabama at Birmingham, Birmingham, Alabama, USA; b Baylor College of Medicine, Houston, Texas, USA; c John T Milliken Department of Medicine, Division of Cardiovascular Disease, Section of Advanced Heart Failure and Transplant, Barnes-Jewish Hospital, Washington University in St. Louis School of Medicine, St. Louis, USA; d Division of Nephrology, Icahn School of Medicine at Mount Sinai, Mount Sinai Hospital, New York, New York, USA; e Harrington Heart & Vascular Institute, University Hospitals Cleveland Medical Center, Case Western Reserve University, Cleveland, Ohio, USA; f Division of Cardiology, Emory University School of Medicine, Atlanta, Georgia, USA; g Department of Cardiology I, Coronary and Peripheral Vascular Disease, Heart Failure Medicine, University Hospital Muenster, Muenster, Germany; h HumanX, Wilmington, Delaware, USA

**Keywords:** clinical outcome, guideline directed medical therapy, HFmrEF, imaging and biomarker, phenotyping, precision medicine

## Abstract

Heart failure with mildly reduced ejection fraction (HFmrEF) is increasingly recognized as a distinct yet heterogeneous phenotype within the heart failure spectrum. Advances in diagnostic tools, including strain imaging, cardiac magnetic resonance, and molecular imaging, together with progress in genomics and biomarker profiling, have contributed to a deeper understanding of the complex pathophysiology of HFmrEF. Although direct, high-quality clinical evidence remains limited due to inconsistent definitions and under-representation in major trials, recent studies and subgroup analyses have offered promising therapeutic options for HFmrEF patients. Given the significant heterogeneity of HFmrEF, future management strategies should focus on precision medicine and individualized care. Integrating advanced imaging, biomarker profiling, genomics, and phenotype-specific assessment will be essential for optimizing patient outcomes. Embracing this precision-based approach promises to redefine care pathways and improve prognosis for this under-recognized patient population.

## Highlights

• Heart failure with mildly reduced ejection fraction represents a clinically significant yet heterogeneous phenotype within the heart-failure spectrum.
• Leverage advanced imaging, biomarkers, and genomic profiling to enhance phenotyping and risk stratification.
• Clinical evidence for heart failure with mildly reduced ejection fraction is increasing, although most data remain extrapolated from heart failure with preserved ejection fraction trials.
• Future progress relies on integrating precision medicine and artificial intelligence to enable data-driven individualized care.

The management of heart failure (HF) is a rapidly evolving field, with significant advances in diagnosis and therapy made over recent years.^1^ Traditionally classified based on left ventricular (LV) ejection fraction (EF) (LVEF), HF management has expanded from a simple binary model of reduced vs preserved ejection fraction to acknowledge the intermediate phenotype: HF with mildly reduced EF (HFmrEF). Defined by an LVEF between 41% and 49%, HFmrEF was formally recognized as a distinct clinical entity in contemporary guidelines, reflecting a growing understanding of its unique pathophysiology and clinical behavior.^1^^,^^2^ Despite being increasingly recognized, HFmrEF remains clinically challenging. LVEF-based classification of HFmrEF, although practical and easily applied in clinical settings, is limited by the inherent variability of LVEF measurements and the narrow range that defines this category. Furthermore, the LVEF of patients with heart failure with reduced EF (HFrEF) or heart failure with preserved EF (HFpEF) may evolve over time into mildly reduced EF range, adding further heterogeneity to the HFmrEF population. Although considerable overlap exists between HFmrEF and both HFrEF and HFpEF populations, emerging evidence from pathophysiological, imaging, and outcome studies increasingly supports the notion that HFmrEF constitutes a distinct HF phenotype. However, high-quality, phenotype-specific clinical evidence for HFmrEF remains scarce, largely due to inconsistent definitions, variability in LVEF thresholds, and under-representation in major randomized controlled trials (RCTs). Furthermore, conventional reliance on LVEF alone fails to capture the complex myocardial dysfunction and diverse phenotypes inherent to this group, underscoring the need for more advanced, precision-based approaches to diagnosis and management.

## Pathophysiology in HFmrEF

HFmrEF represents a pathophysiologically heterogeneous group that exhibits features of both HFpEF and HFrEF. The current understanding indicates that HFmrEF may involve overlapping mechanisms of mild systolic dysfunction, diastolic dysfunction, neurohormonal activation, and systemic inflammation. Despite having a relatively preserved EF compared to HFrEF patients, patients with HFmrEF often demonstrate subtle contractile dysfunction, including reduced global longitudinal strain and diminished contractile reserve. These impairments are typically linked to underlying processes such as ischemic damage, loss of cardiomyocytes, and neurohormonal activation—pathophysiological mechanisms that closely resemble those observed in HFrEF.^3^ Diastolic dysfunction is another hallmark of HFmrEF. Although HFpEF is characterized by pronounced impairments in ventricular relaxation and markedly elevated filling pressures, HFmrEF typically exhibits milder abnormalities, such as delayed relaxation and modest increases in LV stiffness.^4^ In TIME-CHF (Trial of Intensified versus standard Medical therapy in Elderly patients with Congestive Heart Failure) study, echocardiographic findings HFmrEF showed similar patterns of concentric LV hypertrophy compared to HFpEF, albeit to a less degree.^5^ This is different from the HFrEF, where cardiac hypertrophy is primarily eccentric.^6^

HFmrEF patients also exhibit activation of neurohormonal systems such as the renin-angiotensin-aldosterone system and sympathetic nervous system, which contribute to adverse cardiac remodeling and progression of HF.^7^ In a study that measured serum key neurohormonal biomarkers across HFpEF, HFmrEF, and HFrEF patients, 8% of HFmrEF and 10% of HFpEF patients had elevated levels of renin, aldosterone, norepinephrine, and N-terminal pro–B-type natriuretic peptide (NT-proBNP), compared to 21% in HFrEF.^8^ In addition, inflammation plays an important role in HFmrEF. Inflammatory signaling exacerbates fibroblast activation and collagen deposition in the myocardium, promoting stiffness and impaired relaxation causing diastolic dysfunction, whereas cytokine-induced myocyte injury via oxidative stress and apoptosis reduces contractile reserve.^9^ In a biomarker analysis of the COACH (Comparison of Outcomes and Access to Care for Heart Failure) trial, HFrEF was predominantly associated with markers of cardiac stretch and HFpEF with markers of inflammation.^10^ In contrast, HFmrEF showed a unique profile that combined features of both myocardial stretch and inflammation, underscoring the hybrid and overlapping pathophysiological nature of this HF phenotype.^10^

## Epidemiology and etiology

HFmrEF accounts for 13 to 25% of all HF cases across registries and populations.^11^^,^^12^ In the SwedeHF Registry, HFmrEF comprised about 25% of patients, compared with 53% HFrEF and 22% HFpEF.^11^ Patients with HFmrEF generally have demographics between HFrEF and HFpEF, averaging 72 to 75 years of age, with 53% to 55% being male.^13^ HFmrEF has a heterogeneous etiology, reflecting overlap between systolic and diastolic dysfunction. Across registries, ischemic heart disease consistently emerges as the dominant cause, accounting for roughly 40% to 69% of HFmrEF cases.^13^, ^14^, ^15^ In SwedeHF, ischemic etiology was independently associated with both HFrEF and HFmrEF, whereas hypertensive and valvular etiologies predominated in HFpEF.^14^ Hypertension still accounted for approximately 10% to 25% etiology of HFmrEF, followed by valvular and other causes.^14^^,^^15^ In HFmrEF, comorbidities such as atrial fibrillation (AF), hypertension, diabetes, chronic kidney disease (CKD), and obesity are commonly observed, with their distribution showing an intermediate pattern compared to HFrEF and HFpEF.^3^^,^^16^

## Clinical outcome and prognosis of HFmrEF

Outcomes in HFmrEF are generally intermediate between HFrEF and HFpEF, but prognosis depends heavily on etiology. In the ESC-HF registry, 1-year mortality for HFmrEF was 7.6%, compared with 8.8% in HFrEF and 6.3% in HFpEF.^13^ Etiology plays a critical prognostic role: in HFmrEF, hypertensive etiology was linked to a higher risk of HF hospitalization and valvular etiology also conferred an increased hospitalization risk, whereas ischemic etiology was less strongly predictive of hospitalization than in HFrEF. In contrast, ischemic HF remains the most adverse etiology for mortality in HFrEF, whereas in HFmrEF, outcomes are more strongly driven by comorbidity burden.^14^

Up to 39% of HFmrEF patients change phenotype over time, with better survival in those improving to HFpEF and worse outcomes in those progressing to HFrEF.^17^ Conversely, a subset (∼22%) of patients with HFrEF experience partial improvement in LVEF, transitioning into the mildly reduced range or normal EF, now categorized as HF with improved EF (HFimpEF) if they experience more than 10% improvement in EF.^2^^,^^3^ It remains debated whether patients with de novo HFmrEF and those with improved ejection whose LVEF recovers into the mildly reduced range represent the same clinical entity or fundamentally different phenotypes. In contrast to de novo HFmrEF, HFimpEF represents a pathophysiological remission rather than a stable phenotype, characterized by myocardial recovery and reverse remodeling following ischemic, inflammatory, or neurohormonal injury. Data from REDEAL HF (Relationships and Differences Analysis in Heart Failure) trial showed that of 573 patients with baseline HFrEF, 213 patients (37.2%) evolved to HFimpEF during the follow-up of 17 months.^18^ Patients with HFimpEF had significantly lower all-cause mortality (22.1% vs 31.1%), lower cardiovascular (CV) mortality (8.9% vs 16.1%), and fewer CV events (27.2% vs 49.2%) compared to those without EF improvement.^18^ HFmrEF may also encompass patients that progressed from HFpEF, which is not uncommon. In the CHART-2 (Chronic Heart Failure Analysis and Registry in the Tohoku District 2) study, 5.1% of patients with baseline HFpEF transitioned to HFmrEF over time.^3^ SwedeHF registry showed that 13.3% of HFpEF patients experienced a ≥10% decline in LVEF over a median 1.5-year interval.^11^ Predictors of decline in LVEF among patients with HFpEF include high baseline natriuretic peptide levels, ischemic heart disease, higher E/e′, and enlarged LV dimensions as well as lower baseline LVEF within HFpEF range.^11^

## Role of cardiac imaging in HFmrEF

### Echocardiography

Echocardiographic evaluation remains the cornerstone in diagnosing HFmrEF, yet reliance on poses significant challenges. The widely used biplane Simpson method is limited by considerable intraobserver and interobserver variability up to 13% and 21%, respectively.^19^ This is particularly problematic within the narrow 41 to 49% EF range. Moreover, LVEF is also influenced by extrinsic factors such as preload, ventricular geometry, concomitant diastolic dysfunction and right ventricular function.^20^ Therefore, patients with similar EF values may display very different underlying myocardial performance. This inherent variability often results in bidirectional reclassification of patients across HFrEF, HFmrEF, and HFpEF categories, complicating management.^21^ Contrast-enhanced echocardiography and three-dimensional echocardiography have been shown to increase the accuracy of LVEF measurements, and decrease interobserver variability.^22^

Strain imaging provides a more sensitive assessment of myocardial dysfunction than LVEF, with global longitudinal strain (GLS) being the most validated parameter in HF.^23^ Echocardiographic speckle-tracking remains the primary method for GLS assessment because of its accessibility and well-established prognostic value.^23^ In HFmrEF patients, abnormal GLS has been suggested to predict adverse clinical outcome. In a cohort of 273 HFmrEF patients, a GLS worse than −11% was associated with higher mortality and HF hospitalization risk.^24^ Similarly, analysis from PARAGON-HF (Prospective Comparison of ARNI With ARB Global Outcomes in Heart Failure With Preserved Ejection Fraction) study (LVEF ≥45%) showed GLS >−16% was associated with a greater risk for CV death and HF hospitalizations, with no effect modification from LVEF.^25^

Diastolic dysfunction is common in HFmrEF. In a cohort of 1,154 hospitalized HFmrEF patients, 72% showed abnormal filling, although only 16% met American Society of Echocardiography (ASE)/European Association of Cardiovascular Imaging (EACI) grade ≥II diastolic dysfunction.^4^ Moreover, diastolic indices such as E/e′ and left atrial volume index often do not differ meaningfully between HFmrEF and HFpEF.^26^ These findings highlight limitations of traditional diastolic grading schemes, which rely on a few load-dependent Doppler parameters. Consequently, there is a growing need for advanced diastolic assessment that integrates multidimensional markers of myocardial and atrial mechanics. Left atrial (LA) reservoir strain, a load-adjusted measure of atrial compliance and chronic filling pressure, when reduced (LA reservoir strain <18%) in HFmrEF, signifies increased diastolic burden and independently predicts adverse clinical outcomes.^27^ Left atrioventricular (AV) coupling index (LACI), defined as the ratio of LA minimal to LV end-diastolic volume, offers a volumetric measure of AV coupling and has shown incremental prognostic value across EF phenotypes.^28^ Incorporating these advanced indices refines phenotyping, improves risk stratification, and supports more personalized therapy (Table 1).

**Table 1:** Advanced Diagnostic Tools and Biomarkers in HFmrEF and Their Role in Prognostication

Diagnostic Tool	Parameters	Compared to HFrEF and HFpEF	Prognostic Significance in HFmrEF
Echocardiography (2D/3D)	GLS	Intermediate between HFrEF and HFpEF^25^	Impaired GLS (>−16%) indicates subtle systolic dysfunction; GLS >−11% linked to ↑ mortality and HF hospitalization; severity of GLS predicts long-term mortality^24^^,^^25^
	LA reservoir strain	Similar prevalence in HFmrEF and HFpEF^27^	Reduced LASr (<18%) independently predicts CV death and HF hospitalization^27^
	LACI	Intermediate between HFrEF and HFpEF^28^	LACI ≥0.22 predicts all-cause death and HF hospitalization in patients with LVEF <50%^28^
Cardiac MRI	GLS and GCS	FT-CMR GLS and GCS in HFmrEF significantly reduced compared to HFpEF^30^	FT-CMR GLS and GCS predict mortality and reverse remodeling^31^
	T1 mapping	Intermediate between HFrEF and HFpEF^33^	↑ T1 or ECV predicts higher risk of death and HF events^34^
	LGE fibrosis pattern	Intermediate between HFrEF and HFpEF^33^	LGE presence associated with 5-fold ↑ all-cause mortality after adjustment^34^
	LACI	No significant difference compared to HFrEF, lower than HFpEF^33^	LACI>0.31 (3rd tertile) predict all-cause death, HF hospitalization^32^
Cardiac PET/nuclear imaging	Myocardial metabolism	HFmrEF closer to HFrEF pattern with impaired oxidative metabolism^35^	PET markers quantify metabolic inefficiency and correlate with diastolic impairment and worse outcomes.^35^
	Perfusion abnormality	Reduced MFR common in HFmrEF and HFpEF despite no epicardial CAD^37^	Decreased MFR and elevated rest MBF predicts ↑ death or HF hospitalization^38^
Biomarkers	BNP	Intermediate levels (closer to HFpEF)^39^	Above-median NT-proBNP (1,540 pg/mL) associated with 2-fold ↑ risk of death/HF hospitalization;^39^ discharge BNP predicts long-term mortality^41^
	Troponin	Intermediate between HFrEF and HFpEF^40^	hs-cTnI ≥17 ng/L predicts 2-fold ↑ risk of death/LVAD/transplant^42^
Proteomics	SOMAscan	Distinct proteomic profile with immune-modulatory and signal-transduction pathways; shares partial overlap with HFrEF.^44^	Specific protein modules (autophagy, FGFR2, DNA repair) predict adverse events and differentiate biologic subtypes of HFmrEF for precision therapy^43^
Genomics	GWAS, Whole-exome sequencing	Genetic architecture intermediate but closer to HFrEF; 3.8% HFmrEF carry pathogenic cardiomyopathy variants (similar to HFrEF, higher prevalence than HFpEF).^48^	Not available

This table summarizes key imaging modalities and circulating biomarkers used to characterize heart failure with mildly reduced ejection fraction (HFmrEF).

BNP = B-type natriuretic peptide; CAD = coronary artery disease; CV = cardiovascular; ECV = extracellular volume; FGFR2 = fibroblast growth factor receptor 2; FT-CMR = feature-tracking cardiac magnetic resonance; GCS = global circumferential strain; GLS = global longitudinal strain; GWAS = genome-wide association study; HF = heart failure; HFrEF = heart failure with reduced ejection fraction; HFpEF = heart failure with preserved ejection fraction; hs-cTnI = high-sensitivity cardiac troponin I; LA = left atrial; LACI = left atrioventricular coupling index; LASr = left atrial reservoir strain; LGE = late gadolinium enhancement; LVAD; 2D/3D = two-dimensional/three-dimensional; LVEF = left ventricular ejection fraction; NT-proBNP = N-terminal pro–B-type natriuretic peptide; MBF = myocardial blood flow; MFR = myocardial flow reserve; MRI = magnetic resonance imaging; PET = positron emission tomography.

### Cardiac magnetic resonance

Cardiac magnetic resonance (CMR) has become an important tool for strain analysis, offering higher spatial resolution and reproducibility than echocardiography. Commonly used CMR-based strain analysis includes feature-tracking CMR (FT-CMR), CMR tagging, and strain-encoded imaging (SENC/Fast-SENC).^29^ FT-CMR is the most widely adopted in clinical practice, deriving strain parameters like GLS from routine cine images without additional sequences.^29^ A recent study showed GLS and global circumferential strain using FT-CMR were significantly reduced in HFmrEF patients compared to controls and HFpEF, particularly at the subendocardial layer, which has a high diagnostic accuracy for identifying systolic dysfunction and poor HF outcomes.^30^^,^^31^

Beyond strain studies, other CMR-based techniques also provide deeper insight to the cardiac structure and function for patients with HFmrEF, including T1 mapping and extracellular volume for detecting diffuse fibrosis, late gadolinium enhancement (LGE) for identifying focal scar, and LA and LV volumetric index and strain analysis for characterizing AV coupling.^32^, ^33^, ^34^ In 1 study, the presence of LGE lesion was associated with a 5-fold increase in the incidence of all-cause mortality after adjusting for comorbidity for HFmrEF and HFpEF patients.^34^ Compared to HFpEF and HFrEF, patients with HFmrEF exhibited LV dilation, intermediate levels of myocardial fibrosis as detected by T1 mapping and LGE and preserved LA reservoir and conduit function. Despite this preserved LA function, the LACI was significantly impaired, reflecting disrupted mechanical synchrony between the atrium and ventricle. Similarly, LACI was shown to be independently associated with all-cause death or HF hospitalization among HF patients with LVEF<50%.^32^ These advanced CMR parameters enhance structural and functional characterization of HFmrEF beyond LVEF, providing more precise risk stratification.

### Positron emission tomography

Positron emission tomography (PET) provides highly sensitive, noninvasive assessment of myocardial metabolism, perfusion, and inflammation, offering cellular-level insights into substrate-utilization shifts and early dysfunction in HF phenotypes such as HFmrEF and HFpEF.^35^ For example, rubidium-82 PET is commonly used for quantification of myocardial blood flow and myocardial perfusion reserve.^36^ In patients with HFpEF and normal epicardial perfusion on PET imaging, reduced myocardial flow reserve was strongly associated with diastolic dysfunction and impaired LA strain, which supports the important role of coronary microvascular dysfunction.^37^ In a large cohort of 8,089 patients, elevated rest myocardial blood flow and reduced myocardial flow reserve were linked to a significantly higher risk of death or HF hospitalization (HR of 1.39 and 1.7 respectively), with even greater prognostic value in those with HFprEF and HFmrEF.^38^

## Role of biomarker in HFmrEF

### B-type natriuretic peptide and NT-proBNP

B-type natriuretic peptide (BNP) and NT-proBNP are important markers of HF, indicating myocardial wall stress and neurohormonal activation. Elevated BNP levels confirm the presence of hemodynamic congestion and are part of the diagnostic criteria for HF including HFmrEF in guidelines.^1^ In HFmrEF, their levels are usually between those of HFrEF and HFpEF, but generally closer to HFpEF.^39^^,^^40^ Study from SwedeHF has shown the elevation of NT-proBNP level above the median level (1,540 pg/mL for HFmrEF) is associated with a 2-fold increased risk of composite death and HF hospitalization.^39^ Similarly, the discharge BNP level has been shown to be associated with increased long-term mortality after acute HF hospitalization (HR: 1.76 and C-index: 0.68 for HFmrEF).^41^

### Troponin

High-sensitivity cardiac troponins (hs-cTnI or hs-cTnT) capture ongoing myocardial injury and have emerged as strong prognostic markers in HFmrEF. In biomarker profiling studies, HFmrEF patients show troponin concentrations that lie between those of HFrEF and HFpEF. In 1 study comprised of 520 HFrEF and HFmrEF patients, hs-cTnI ≥17 ng/L identified those at markedly higher 2-year risk of death, heart transplantation, or left ventricular assist device implantation (OR: 2.13), independent of NT-proBNP and clinical parameters.^42^ The study also showed that adding hs-cTnI to NT-proBNP significantly improved prognostic discrimination (area under the curve [AUC]: 0.82 vs 0.78), underscoring the complementary role of troponin in refining risk stratification in HFmrEF.^42^

### Proteomics

Proteomics have emerged as a powerful tool in HF, enabling high-throughput profiling of circulating and tissue proteins that reflect the complex biological processes underlying the HF phenotypes.^43^^,^^44^ SOMAscan-based analysis of over 1,000 circulating proteins among different HF phenotypes demonstrated that HFrEF, HFmrEF, and HFpEF possess distinct proteomic signatures that correspond to divergent biological pathways. HFrEF was predominantly associated with growth-factor signaling, extracellular matrix remodeling, and cell-survival pathways, whereas HFpEF showed enrichment of inflammatory, angiogenic, and metabolic dysregulation pathways. In contrast, HFmrEF exhibited a unique proteomic profile characterized by significant activation of immune-modulatory and signal transduction.^44^ In a more recent study of 442 HF patients (including 125 HFmrEF patients) that investigated 4,670 proteins, HFmrEF shared substantial proteomic overlap with HFrEF, particularly across pathways related to RNA processing, DNA repair, fibroblast growth factor receptor 2 signaling, and autophagy, indicating persistent cellular stress and structural remodeling that is more different from HFpEF.^43^ Collectively, these proteomic insights underscore HFmrEF as a biologically hybrid yet distinct entity and highlight the potential of proteomics for precision phenotyping and target therapeutic development.

### Genomics in HFmrEF

Genomics is central to precision medicine in HF by enabling earlier diagnosis, improved risk stratification, and personalized treatment. Although significant progress has been made through large-scale genome-wide association and multiomics studies, these efforts have primarily focused on HFpEF and HFrEF.^45^, ^46^, ^47^ To date, no study has analyzed HFmrEF as a distinct category. Instead, patients within this range are typically grouped with HFrEF (defined as LVEF <50%) or excluded entirely.^46^ Consequently, the genetic architecture and molecular drivers of HFmrEF remain poorly defined. Current understanding relies largely on extrapolation from HFrEF and HFpEF findings, where key loci such as *BAG3*, *FLNC*, *TTN*, and *CDKN1A* highlight overlapping pathways in sarcomere integrity, inflammation, metabolism, and fibrosis.^46^ Furthermore, studies of large-scale consortia such as HERMES have identified over 100 genetic loci linked to HF, highlighting disease mechanisms related to sarcomere integrity, protein homeostasis, metabolism, and inflammation.^45^ HFpEF risk was enriched for genetic variants linked to hypertension, obesity, and type 2 diabetes, whereas HFrEF and HFmrEF, by contrast, showed stronger associations with variants affecting myocardial structure and function, such as those in TTN (titin), FLNC, BAG3 (Bcl2-associated athanogene 3), and MYBPC3 (myosin-binding protein C).^46^ Other than large genome-wide association studies, genetic study from clinical trials and registries also offers opportunities to investigate the genetics association of HFmrEF. A whole-exome sequencing study of >5,900 patients with HF from the CHARM (Candesartan in Heart failure-Assessment of Reduction in Mortality and morbidity) and CORONA (Controlled Rosuvastatin Multinational Trial in Heart Failure) trial and U.K. BioBank have found that up to 3.8% of HFmrEF patients possess pathogenic cardiomyopathy variants such as TTN, MYBPC3, and FLNC, a rate comparable to HFrEF and higher than HFpEF.^48^ Future studies integrating genotype, transcriptomic, and proteomic data with refined phenotyping are needed to clarify whether HFmrEF possesses a unique genomic signature or reflects a transitional phenotype along the HF continuum.

## Guideline-directed medical therapy for HFmrEF

### Angiotensin receptor neprilysin inhibitor

Angiotensin receptor neprilysin inhibitors (ARNIs) combines a neprilysin inhibitor (sacubitril) with an angiotensin receptor blocker (valsartan), with sacubitril enhancing natriuretic peptide activity while valsartan counteracts renin-angiotensin-aldosterone system activation. In HFrEF, ARNI therapy has been firmly established as a cornerstone treatment following the landmark PARADIGM-HF (Prospective Comparison of ARNI with ACEI to Determine Impact on Global Mortality and Morbidity in Heart Failure) trial, which demonstrated significant reductions in CV mortality and HF hospitalizations compared to traditional angiotensin-converting enzyme inhibitors.^49^ However, the following PARAGON-HF (Prospective Comparison of ARNI With ARB Global Outcomes in Heart Failure With Preserved Ejection Fraction) trial failed to meet its primary endpoint in patients with preserved EF (45% or higher).^50^ Notably, Subgroup analyses suggested a potential benefit of ARNI therapy among patients with lower EF (LVEF 45%-57%) and in women.^50^ A prespecified participant-level pooled analysis of PARAGLIDE-HF (Prospective comparison of ARNI with ARB Given following stabiLization In DEcompensated HFpEF) and PARAGON-HF trial further demonstrated that sacubitril/valsartan was associated with significant reduction of composite outcome of total worsening HF events and CV death, with strongest benefit in patients with LVEF ≤60%.^51^ In a meta-analysis of 4 RCTs including 3,375 patients with HFmrEF and HFpEF, sacubitril/valsartan resulted in improvements in the HF outcomes of the Kansas City Cardiomyopathy Questionnaire (KCCQ) Clinical Summary Score, the NYHA functional class, and the composite outcome of hospitalization for HF and CV death vs valsartan.^52^ These findings strongly suggest the potential benefit of ARNI for patients with HFmrEF (Table 2).

**Table 2:** Selected Landmark HF Trials That Included HFmrEF Patients

	Intervention	EF Criteria	Total Patient Number/% HFmrEF	Main Outcome	HFmrEF Specific Outcome
ARNI/ARB/ACEI					
PARAGON-HF	Sacubitril/valsartan vs valsartan	LVEF ≥45%	N = 4,822/∼25%	Neutral on primary composite (total HFH + CV death), ↓ total HFH (RR: 0.85)	Greater benefit in EF 45-57% and in women^50^
PARAGLIDE-HF	Sacubitril/valsartan vs valsartan	LVEF >40%	N = 467/∼23%	↓NT-proBNP at weeks 4–8 (ratio 0.85) Neutral on secondary endpoint (CV death, HFH, HF emergency room visit, Δ NT-proBNP)	NT-proBNP reduction and clinical benefit greater at lower EF^51^
BB					
SENIORS	Nebivolol vs placebo	No upper EF cutoff	N = 2,128/-	↓Composite of all-cause death or CV hospital admission (HR: 0.86)	Post hoc analyses: no significant heterogeneity by EF
MRA					
TOPCAT	Spironolactone vs placebo	LVEF ≥45%	N = 3,445/∼30%	Neutral on primary composite (CV death, cardiac arrest, HFH); ↓ HFH (HR 0.83)	Benefit more pronounced in lower EF and Americas cohort^60^
FINEARTS-HF	Finerenone vs placebo	LVEF ≥40%	N = 6,000/∼30%	↓ Composite of total HF events + CV death (RR: 0.84)	Effect consistent across EF including 40–49%^61^
SGLT2i					
EMPEROR-Preserved	Empagliflozin	LVEF >40%	N = 5,988/∼33%	↓ Composite (CV death or HFH) (HR 0.79), driven by ↓ HFH	EF 41–49% showed greater absolute risk reduction^66^
DELIVER	Dapagliflozin	LVEF >40%	N = 6,263/∼18%	↓ Composite (CV death or worsening HF) (HR: 0.82), driven by ↓ HFH	Effect consistent across EF^65^
GLP-1RA					
STEP-HFpEF	Semaglutide vs placebo	LVEF ≥45%	N = 529/∼15%–20%	↑ KCCQ-CSS; ↑ 6MWD; large weight loss; ↓ NT-proBNP; fewer HF urgent/hospital events (HR: 0.58)	Not available
STEP-HFpEF DM	Semaglutide vs placebo	LVEF ≥45%	N = 616/∼15%–20%	↑ KCCQ-CSS; ↑ 6MWD; large weight loss; signal toward fewer HF events (HR: 0.40)	Not available
SELECT	Semaglutide vs placebo	No EF cutoff (includes patient with HF history)	N = 17,604/∼5%	↓ Composite of CV death, nonfatal MI, or nonfatal stroke (HR: 0.80)	Effect consistent in EF ≥50% and EF<50%^76^
Vericiguat					
VICTORIA	Vericiguat vs placebo	LVEF ≥45%	N = 5,050/∼13%	↓Composite of CV death or first HF hospitalization (HR: 0.90)	Meta-analysis of both trials showed no significant benefit was observed in the HFpEF cohort (LVEF ≥45%) for primary outcomes, cardiovascular death^85^
VITALITY-HFpEF	Vericiguat vs placebo	LVEF ≥45%	N = 789/∼20%	No significant improvement in physical limitation score (KCCQ PLS) at 24 wk	
CCM					
FIX-HF-5	CCM + GDMT vs GDMT alone	LVEF ≤45%	N = 428/∼15%	↑Peak Vo_2,_ ↑QoL (MLWHFQ), ↑6MWD, ↑NYHA functional class	Not available
FIX-HF-5C	CCM + GDMT vs GDMT alone	LVEF 25-45%	N = 160/∼35%	↑Peak Vo_2_↑QoL (MLWHFQ), ↑6MWD, ↑NYHA functional class	Subgroup analysis of LVEF 35%–45% showed greater gains in functional capacity and quality of life than those with lower EF^87^
M-TEER					
COAPT	MitraClip + GDMT vs GDMT alone	LVEF 20-50%	N = 614/∼20%	↓HFH (HR: 0.53), ↓All-cause death (HR: 0.62)	Treatment effect consistent across EF strata (20%-50%)^113^
RESHAPE-HF2	MitraClip + GDMT vs GDMT alone	LVEF 20-50%	N = 505/∼25%	↓ Composite of total HFH + CV death (RR: 0.63)	Treatment effect consistent across EF strata (20%-50%)^114^

This table summarizes major randomized controlled trials that enrolled patients with HFmrEF, highlighting the intervention, ejection fraction criteria, proportion of HFmrEF participants, and main clinical outcomes.

ACEI = angiotensin-converting enzyme inhibitor; ARB = angiotensin receptor blocker; ARNI = angiotensin receptor neprilysin inhibitor; BB = beta-blocker; CCM = cardiac contractility modulation; CSS = Clinical Summary Score; EF = ejection fraction; GDMT = guideline-directed medical therapy; GLP-1RA = glucagon-like peptide-1 receptor agonist; HFH = heart failure hospitalization; KCCQ = Kansas City Cardiomyopathy Questionnaire; MI = myocardial infarction; MRA = mineralocorticoid receptor antagonist; M-TEER = transcatheter mitral valve edge-to-edge repair; MWD = minute walk distance; PLS = Physical Limitation Score; QoL = quality of life; SGLT2i = sodium-glucose cotransporter 2 inhibitor; other abbreviations as in Table 1.

### Beta-blocker

Beta-blockers (BBs) function as neurohormonal antagonists by blocking beta-adrenergic receptors, thereby counteracting the deleterious effects of sympathetic overactivation in HF. In patients with HFrEF, BBs have consistently demonstrated mortality benefits, serving as a cornerstone therapy within guideline-directed medical therapy (GDMT).^1^ In contrast, the benefit of BB for HFpEF is less clear, and potential harm has been suggested possibly due to worsening chronotropic incompetence and diastolic dysfunction.^53^^,^^54^ For the HFmrEF patient, the effect of BB is also less consistent but does not appear to cause significant harm.^54^ Data from the large PINNACLE registry indicated that the BB use was associated with a lower risk of both HF hospitalization and death for HFmrEF patients whereas a higher risk for HFpEF patients.^55^ In contrast, data from DELIVER (Dapagliflozin Evaluation to Improve the Lives of Patients with Preserved Ejection Fraction Heart Failure) trial (EF >40%) showed BB was associated with a lower risk of primary outcome in covariate-adjusted model (HR: 0.7), which is not modified by the LVEF categories.^56^ In a recent pooled analysis of individual patient data from 4 major randomized trials (I-Preserve, TOPCAT [Treatment of Preserved Cardiac Function Heart Failure with an Aldosterone Antagonist Trial], PARAGON-HF, and DELIVER) that included HFmrEF and HFpEF, nonadjusted HR for the primary outcome did not differ between BB users and nonusers, with potential benefit noticed for the patient with AF.^57^ In the same study, BB use was associated with lower CV death and HF hospitalization among HFmrEF but not HFpEF.^57^ Lastly, an individual patient-level meta-analysis of 4 randomized trials (REBOOT [Beta-Blockers after Myocardial Infarction without Reduced Ejection Fraction], BETAMI [BEtablocker Treatment After acute Myocardial Infarction in revascularized patients without reduced left ventricular ejection fraction], DANBLOCK [Danish trial of beta-blocker treatment after myocardial infarction without reduced ejection fraction], and CAPITAL-RCT [Carvedilol Post-Intervention Long-Term Administration in Large-scale Randomized Controlled Trial]) demonstrated that in patients with acute myocardial infarction (MI) and mildly reduced EF (without overt HF), BB therapy significantly reduced the composite outcome of all-cause death, recurrent MI, or HF.^58^ In summary, BB use for HFmrEF appears to be safe and offers potential benefit, especially for those with compelling indications such as recent MI, AF, or ventricular tachycardia.

### Mineralocorticoid receptor antagonist

Mineralocorticoid receptor antagonists (MRAs), such as spironolactone and eplerenone, play a well-established role in HFrEF.^1^ TOPCAT trial first evaluated the role of MRAs for HFpEF (defined as EF ≥45%) but did not significantly reduce the primary composite endpoint of CV death, aborted cardiac arrest, or HF hospitalization. However, it did lead to a significant reduction in HF hospitalizations.^59^ Importantly, post hoc analysis suggested that the benefits were most evident in patients with lower-end LVEF of 45% to 50%.^60^ More recently, analysis of contemporary clinical trials provided more insight into the role of MRA on HFmrEF. The FINEARTS-HF (Finerenone Trial to Investigate Efficacy and Safety Superior to Placebo in Patients with Heart Failure) trial demonstrated that finerenone, a novel nonsteroidal MRA, significantly reduced the risk of total HF events and CV death in patients with HFmrEF and HFpEF, with an acceptable safety profile.^61^ A large individual patient-level meta-analysis incorporating data from RALES (Randomized Aldactone Evaluation Study), EMPHASIS-HF (Eplerenone in Mild Patients Hospitalization and Survival Study in Heart Failure), TOPCAT, and FINEARTS-HF showed that MRAs consistently reduced HF hospitalizations across the EF spectrum, although the mortality benefit remained more pronounced in HFrEF.^62^ Differential response to MRAs among HF patients also suggests underlying heterogeneity within the HFmrEF population. In a study of phenomapping analyses from TOPCAT showed that spironolactone significantly reduced adverse outcomes in obese, diabetic patients with advanced symptoms, whereas other phenotypes derived little benefit.^63^ Similarly, in FINEARTS-HF trial, when body mass index (BMI) was examined as continuous variable, the benefit of the finerenone appears to be greater in patient with higher BMI.^64^ These findings support a precision medicine approach, using phenotype-guided strategies to optimize MRA therapy in HFmrEF.

### Sodium-glucose cotransporter 2 inhibitor

Sodium-glucose cotransporter 2 inhibitors (SGLT2is) have emerged as a cornerstone therapy in HF management, initially recognized for their benefits in HFrEF and now increasingly validated in HFmrEF and HFpEF.^1^ For patients with HFmrEF, landmark trials such as EMPEROR-Preserved (LVEF >40%) and DELIVER (LVEF >40%) has demonstrated that SGLT2i significantly reduce the risk of worsening HF events and CV death, with benefits comparable to those seen in HFrEF.^65^^,^^66^ In addition, pooled analysis of major trials confirms that the efficacy of SGLT2i spans the entire EF spectrum without significant attenuation in the HFmrEF range.^67^ In addition to its role in HF, SGLT2i has been proven to benefit patients with type 2 diabetes mellitus,^68^ CKD,^69^ therefore SGLT2i may offer additional value for HFmrEF patients with these comorbidities. Mechanistically, SGLT2i also exerts beneficial effects including reverse cardiac remodeling, antiarrhythmic effect, improvement in diastolic function and metabolic modulation, providing a rationale for precision medicine strategies that tailor therapy toward HFmrEF patients with specific phenotypic profiles.^70^

### Glucagon-like peptide-1 receptor agonist

Glucagon-like peptide-1 receptor agonists (GLP-1RAs), including semaglutide and tirzepatide, were originally developed for the management of type 2 diabetes and obesity.^71^ These agents mimic endogenous GLP-1 hormone activity, promoting glucose-dependent insulin secretion, appetite suppression, and weight loss.^71^ In recent years, 2 landmark trials STEP-HFpEF (Semaglutide Treatment Effect in People with obesity and Heart Failure with preserved ejection fraction) and STEP-HFpEF-DM have demonstrated that semaglutide significantly improves HF-related symptoms, physical limitations, and exercise capacity, while also reducing body weight and systemic inflammation in patients with obesity-related HFpEF (LVEF ≥45%).^72^^,^^73^ These benefits were observed regardless of diabetic status and were accompanied by a reduction in loop diuretic use, suggesting a primary decongestive effect and potential disease-modifying role for HF patients.^74^ Indeed, the SELECT trial suggested semaglutide was associated with reduced major adverse CV events by 20% in patients with obesity and established CV disease, without diabetes.^75^ A prespecified study of SELECT trial patient with HF history (LVEF <50% or ≥50%) showed the benefit of semaglutide remains significant in this population, regardless of the LVEF.^76^ Although direct evidence in HFmrEF remains limited, a substantial proportion of patients in these trials fall within this category. Notably, a recent patient-level meta-analysis combining data from the SELECT, FLOW, STEP-HFpEF, and STEP-HFpEF-DM trials that includes HFmrEF and HFpEF patients demonstrated that semaglutide significantly reduced the composite endpoint of CV death or worsening HF events, primarily driven by a reduction in HF hospitalizations.^77^ Beyond obesity and diabetes, GLP-1RA may also confer renal protection for CKD patients. The FLOW trial, specifically designed to assess kidney outcomes, showed that semaglutide reduced the risk of clinically important kidney endpoints by 24% compared to placebo in patients with diabetes and established CKD.^78^ Together, these findings position GLP-1RAs as a promising individualized therapy for HF, particularly in patients with obesity, diabetes, or CKD.^79^

### Vericiguat

Vericiguat is a soluble guanylate cyclase stimulator, enhances cyclic guanosine monophosphate production by directly stimulating soluble guanylate cyclase and sensitizing it to nitric oxide, thereby improving vascular tone, reducing fibrosis, and countering myocardial dysfunction.^80^ Landmark VICTORIA (Vericiguat Global Study in Subjects with Heart Failure with Reduced Ejection Fraction) trial has demonstrated that vericiguat, in addition to standard therapy, significantly reduced CV death or HF hospitalization in patients with HFrEF (LVEF <45%) and recent worsening HF.^81^ The recent VICTOR (Vericiguat Global Study in Participants with Chronic Heart Failure) trial further highlights its role in stable HF patient with its benefit of reducing long-term mortality.^82^ In contrast to HFrEF patients, the role of vericiguat for patients with HFmrEF and HFpEF remains unclear. SOCRATES-PRESERVED (SOluble guanylate Cyclase stimulatoR in heArT failurE patientS with PRESERVED EF) trial evaluated vericiguat in patients with worsening chronic HFpEF (EF ≥45%, NYHA functional class II-IV) and found although vericiguat did not significantly improve NT-proBNP or LA volume compared to placebo, it was associated with a significant improvement in quality of life measured by KCCQ score.^83^ However, the VITALITY-HFpEF (Vericiguat to Improve Physical Functioning in Daily Living Activities of Patients With Heart Failure and Preserved Ejection Fraction) trial which includes larger numbers of patients with symptomatic HFpEF (defined as EF ≥45%, NYHA functional class II-III) failed to reach its primary (change in the Physical Limitation Score of KCCQ) or secondary endpoint (change in 6-minute walk distance).^84^ A pooled meta-analysis of VICTORIA (LVEF <45%) and VITALITY-HFpEF (LVEF ≥45%) showed clear benefit of vericiguat in HFrEF (especially in those with EF <24%), but not among HF patients with EF >45% for primary outcome (CV death or first HF hospitalization).^85^ Taking together, the above findings consistently indicate that vericiguat did not produce significant improvements in surrogate biomarkers or clinical outcomes in patients with LVEF ≥45%. Therefore, there is insufficient evidence to support its routine use in this population, and the therapeutic role of vericiguat in HFmrEF and HFpEF patient warrants further investigation in future clinical trials.

### Cardiac contractility modulation

Cardiac contractility modulation (CCM) is an emerging device therapy designed to address the unmet therapeutic needs in HF patients who remain symptomatic despite optimal medical management. CCM delivers nonexcitatory electrical signals during the absolute refractory period of the cardiac cycle, improving myocardial calcium handling, reversing maladaptive gene expression, and promoting reverse remodeling without increasing myocardial oxygen consumption.^86^ Landmark trials such as FIX-HF-5 (Evaluate Safety and Efficacy of the OPTIMIZER System in Subjects With Moderate-to-Severe Heart Failure) and FIX-HF-5C (Confirmatory Randomized Trial Evaluating the Optimizer System) established CCM’s efficacy in HFrEF and NYHA functional class III demonstrating improvements in functional capacity, quality of life, and exercise tolerance among patients with LVEF between 25% and 45% who were not candidates for cardiac resynchronization therapy.^87^^,^^88^ Although there is no dedicated clinical trial targeting HFmrEF yet, there is growing evidence that CCM might be beneficial for HFrEF with higher end EF and HFmrEF patients. Subgroup analyses from the FIX-HF-5C trial demonstrated that patients with LVEF between 35% and 45% experienced greater improvements in functional capacity and quality of life compared to those with lower EF.^87^ Registry data from the CCM-REG study indicated that patients with EF 35% to 45% had survival rates better than predicted by the Seattle Heart Failure Model, supporting CCM's effectiveness in this group.^89^ In addition, CCM has been shown in improve LVEF and GLS among recipients.^90^ Although CCM is primarily designed for patients with lower EF, its therapeutic role may extend beyond LVEF improvement because of its pleiotropic effects on cardiac remodeling, cellular energetics, and inflammation.^91^ These mechanisms collectively support continued investigation of CCM use among patients with HFmrEF, offering a promising nonpharmacologic therapeutic option for those who remain symptomatic despite optimal medical therapy.^91^

## Special considerations of selected comorbidities in HFmrEF

### Obesity

HFmrEF shares several phenotypic traits with HFpEF, including a high prevalence of metabolic comorbidities such as obesity, diabetes, and hypertension.^3^ Obesity HFpEF phenotype was characterized by increased plasma volume, concentric biventricular remodeling, epicardial adiposity, and elevated filling pressures.^92^ CMR-based phenotyping using *native T1 mapping and strain imaging has demonstrated* preserved EF but abnormal GLS and elevated T1 among HFpEF patients with obesity.^93^ Biomarker analysis further identifies distinct inflammatory signatures in obesity-related HF, with paninflammatory clusters exhibiting the most adverse biomarker profile and the worst clinical outcomes.^94^

Clinically, BMI and HF outcomes follows a U-shaped or J-shaped pattern, with both low body weight and severe obesity are associated with negative outcomes, whereas mildly obese individuals generally experience more favorable prognoses. In a multivariable adjusted analysis of 1,832 hospitalized HFmrEF patient, each 1 kg/m^2^ increase in BMI corresponded to a 4% relative reduction in mortality risk (HR: 0.96), without affecting HF rehospitalization.^95^ In DELIVER trial (LVEF>40%), the relationship of CV death and all-cause death and BMI also observe a U-shape pattern (class I obesity being lowest risk) where HF hospitalization risk is proportional to the obesity severity.^96^ These differing associations between BMI and fatal vs nonfatal outcomes may reflects greater metabolic reserve and better treatment tolerance in overweight and mildly obese patients, whereas extreme obesity increases congestion and inflammation, raising rehospitalization risk.^97^ Newer anthropometric markers such as waist to height ratio and epicardial adipose tissue outperformed BMI in predicting adverse HF outcome among HFpEF and HFmrEF patients in a strong and linear association.^92^^,^^98^ Effect of weight loss on HF also presents a complex picture, where weight reduction does not uniformly translate to improved outcome. Although intentional weight loss through healthy lifestyle modification, GLP-1RA,^73^ or bariatric surgery^99^ has been associated with improved symptoms, functional capacity, and reduced hospitalization in obese patients with HFmrEF and HFpEF, unintentional or disease-driven weight loss often reflects advanced disease and is associated with increased morbidity and mortality.^100^

### Atrial fibrillation

AF is another important comorbidity in HFmrEF due to its high prevalence, adverse prognostic impact, and intertwined pathophysiology with HF.^101^ Data from SwedeHF Registry suggested approximately 60% prevalence of AF among HFmrEF, lower than HFpEF and higher than HFrEF.^102^ AF was equally associated with an increased risk of mortality and HF hospitalization across all LVEF groups, including HFmrEF.^102^ Landmark trials such as CASTLE-AF (Catheter Ablation vs. Standard Conventional Treatment in Patients With LV Dysfunction and AF) and AATAC (Ablation versus Amiodarone for Treatment of persistent Atrial fibrillation in patients with Congestive heart failure and an implanted device) have demonstrated the rhythm control using catheter ablation was associated with improved mortality and reduced unplanned hospitalization among patient with HFrEF.^103^^,^^104^ In contrast, the benefit of rhythm control in HFmrEF patients has not been well established due to the lack of dedicated clinical trial for this population. In a prespecified analysis of EAST-AFNET4 (Early Treatment of Atrial Fibrillation for Stroke Prevention Trial) trial of HF patients (26.9% HFmrEF), early rhythm control therapy was not associated with more improvement in EF compared to usual care among patients with HFrEF and HFmrEF, although it reduces mortality and HF hospitalizations in all HF groups.^105^ Similarly, another prespecified subgroup analysis from CABANA (Catheter Ablation Versus Anti-arrhythmic Drug Therapy for Atrial Fibrillation) trial (11.7% HFmrEF) also suggested a significant reduction in primary endpoint and all cause-mortality, regardless of LVEF.^106^ Conversely, a meta-analysis of 12 RCTs with 2,465 HF patients failed to show significant benefit of catheter ablation for HFpEF (defined as EF ≥40%) patients.^107^ Given the uncertain benefits of rhythm control and the intrinsic heterogeneity of HFmrEF, a personalized management approach is warranted for HFmrEF patients with AF. With the increased availability of smart wearables for rhythm monitoring and new clinical tools to stratify both the risk and severity of AF, key management decisions such as the choice between rhythm vs rate control strategies and the initiation of anticoagulation can now be further tailored to individual patient needs.^108^

### Mitral valve regurgitation

Functional mitral regurgitation (FMR) is common across the HF spectra, including HFmrEF. Moderate or severe secondary mitral regurgitation affects up to 30% of HF patients and is most prevalent in HFrEF, followed by HFmrEF and HFpEF.^13^ In a 2024 registry of 890 HFmrEF patients, MR severity correlated with worse outcomes: 1-year composite endpoint (death or HF readmission) occurred in 23.5%, 32.9%, and 36.5% for none/mild, moderate, and moderate-to-severe MR, respectively.^109^ Moderate and severe MR independently predicted mortality and rehospitalization (HR: 1.38 and 1.55, respectively).^109^ MR in HFmrEF is primarily functional with frequent coexistence of atrial and ventricular MR, reflecting its mixed pathophysiology.^110^ Despite its prognostic significance, direct therapeutic evidence in HFmrEF remains limited. Optimization of GDMT is fundamental, as these agents promote LV reverse remodeling and subsequently reduce MR severity.^110^ In PRIME (Pharmacological Reduction of Functional, Ischemic Mitral Regurgitation) study (LVEF 25%-50%), ARNI was associated with significant reduction in effective regurgitant orifice and regurgitant volume.^111^ Device treatment such as mitral valve transcatheter edge-to-edge repair (M-TEER) can be considered in refractory or advanced FMR patients. In COAPT (Cardiovascular Outcomes Assessment of the MitraClip Percutaneous Therapy for Heart Failure Patients with Functional Mitral Regurgitation) trial, M-TEER significantly improved all-cause death and HF hospitalization in patient with severe FMR and LVEF of 20 to 50%.^112^ With post hoc analyses showing consistent benefit irrespective of LVEF.^113^ Recent RESHAPE-HF2 (Randomized Investigation of the MitraClip Device in Heart Failure: 2nd Trial in Patients with Clinically Significant Functional Mitral Regurgitation) that targets HF patient with LVEF of 20 to 50% and moderate to severe FMR, M-TEER was also shown to significantly reduce of HF hospitalization and CV death, further expanding its indication.^114^ Overall, these data position FMR as a key, modifiable target in HFmrEF, emphasizing individualized optimization of GDMT and selective application of M-TEER as part of a precision medicine approach to improve patient outcomes.

## Current challenge

HFmrEF represents a clinically heterogeneous population, reflecting pathophysiological overlap between HFrEF and HFpEF phenotypes. Despite its recognition as a distinct entity in recent HF classifications, high-quality clinical evidence specific to HFmrEF remains limited. This is largely due to inconsistent definitions across studies, varying LVEF cutoffs used to define the HFmrEF group, and under-representation in major randomized clinical trials. As a result, current treatment recommendations for HFmrEF are primarily extrapolated from subgroup analyses of HFrEF or HFpEF populations with limited evidence strength.^1^ Diagnostic challenges continue to impede the adoption of phenotype-driven approaches, as conventional reliance on LVEF fails to capture the nuanced pathophysiological features of this group. Advanced imaging modalities such as strain imaging and tissue characterization are not yet routinely implemented in clinical practice and are largely confined to specialized, high-volume centers.^29^ In addition, the high burden of comorbidities common in HFmrEF patients underscores the need for a more individualized, precision-based approach when selecting treatment strategies.

### Future direction

Future management of HFmrEF must pivot toward a precision medicine approach, integrating multimodal imaging, biomarker profiling, and genomic characterization to address the heterogeneity of this condition. Advanced imaging tools, such as GLS, CMR-based strain, T1 mapping, and PET-derived myocardial flow reserve, enable more nuanced assessment of cardiac structure and function and should guide risk stratification and therapeutic decision-making.^23^ Genomic testing, including identification of pathogenic variants and polygenic risk scores, offers potential for early diagnosis and individualized therapy, particularly for patients with familial or arrhythmogenic predispositions.^45^ Moreover, targeted phenotyping such as obesity and inflammation-related HF may identify subgroups amenable to emerging treatments such as GLP-1RA or anti-inflammatory strategies (Table 3).

**Table 3:** GDMT Recommendation for HFmrEF Patients and Precision Medicine Consideration

GDMT	Evidence Strength in HFmrEF	HF Guideline Recommendations	Precision Medicine Consideration in HFmrEF
ARNI/ARB/ACEI	+++	AHA/ACC/HFSA 2022: ARNI/ACEI/ARB Class IIb for HFmrEF; ESC 2023: ARNI/ACEI/ARB Class IIb (symptomatic, esp. EF at lower end)	Useful in hypertensive/ischemic phenotypes; can lessen functional MR (PRIME);^111^ improves renal outcome
BB	++	AHA/ACC/HFSA 2022: Class IIb (HFmrEF); ESC 2023: Class IIb	No apparent harm, benefit strongest when another indication exists (recent MI, AF with rate control, VT).^57^^,^^58^
MRA	+++	AHA/ACC/HFSA 2022: Class IIb (HFmrEF); ESC 2023: Class IIb (consider esp. at lower EF)	Obesity/DM phenotypes: stronger signal (TOPCAT, FINEARTS-HF);^63^^,^^64^ improves renal outcome
SGLT2i	++++	ESC 2023: Class I (HFmrEF); AHA/ACC/HFSA 2022: Class IIa (HFmrEF)	First-line when DM or CKD present (adds glycemic/renal protection);^68^^,^^69^ improves renal outcome; weight-neutral to modest loss; may reduce AF events
GLP-1RA	++	No recommendation for HFmrEF	Strong consideration in obese ± T2DM phenotypes (for HF symptom relief/weight loss); may also improve renal outcome,^78^ combine with SGLT2i for DM/CKD when feasible.
Vericiguat	+	No recommendation for HFmrEF	Consider in HFmrEF with worsening events and with EF in the lower end (near 40%);^85^ no CKD/obesity-specific signal.
CCM	+	In selected symptomatic NYHA functional class III with LVEF 25%–45% not CRT candidate	Consider in persistent symptoms despite GDMT, especially EF 41%–45%;^87^ no obesity/DM-specific signal.
M-TEER	++	ACC/AHA VHD 2020: TEER class IIa in COAPT-like FMR (NYHA functional class II–IV, severe FMR, suitable anatomy, LVEF 20%-50% etc)	Consider TEER if persistent moderate to severe FMR.^113^^,^^114^ Elevated procedural risk for CKD/obesity patient

This table summarizes the current evidence strength, major society guideline recommendations, and phenotype-specific precision medicine considerations for key pharmacologic and device-based therapies in HFmrEF, integrating comorbidities and individualized treatment approaches.

ACC = American College of Cardiology; AF = atrial fibrillation; AHA = American Heart Association; CKD = chronic kidney disease; CRT = cardiac resynchronization therapy; DM = diabetes mellitus; ESC = European Society of Cardiology; FMR = functional mitral regurgitation; HFSA = Heart Failure Society of America; T2DM = type 2 diabetes mellitus; VHD = valvular heart disease; VT = ventricular tachycardia; other abbreviations as in Table 1 and 2.

However, integrating these diverse diagnostic and prognostic modalities into a coherent management framework remains challenging. Artificial intelligence (AI) and big-data analytics are poised to play a transformative role by enabling large-scale integration of clinical, imaging, biomarker, and genomic data to refine phenotyping, improve outcome prediction, and support individualized decision-making. For example, in a cohort of *424 HFmrEF patients*, machine-learning models using 67 clinical and laboratory variables achieved an *AUC of 0.92* for mortality prediction, outperforming traditional regression and demonstrating the power of data-driven prognostication.^115^ AI can be also used in predicting response to GDMT. A machine learning–based analysis of 294 patients with HFrEF and HFmrEF treated with sacubitril/valsartan integrated XGBoost and clustering methods to predict LVEF improvement, achieving an *AUC of 0.70* in the HFmrEF subgroup and identifying *renal function, QRS duration, and ischemic etiology* as the strongest predictors of reverse remodeling.^116^ Taking together, AI offers a great opportunity to operationalize a true precision medicine approach in the heterogeneous population of HFmrEF. By integrating multimodal data such as imaging parameters, biomarkers, clinical variables, and genomic information, AI-driven models can uncover latent phenotypes, predict therapeutic responsiveness, and personalize treatment strategies. Ultimately, these tools hold promise to move HFmrEF management beyond conventional EF-based definitions toward *mechanism- and phenotype-guided precision therapy*, improving both patient selection and clinical outcomes (Central Illustration).

**Central Illustration:**
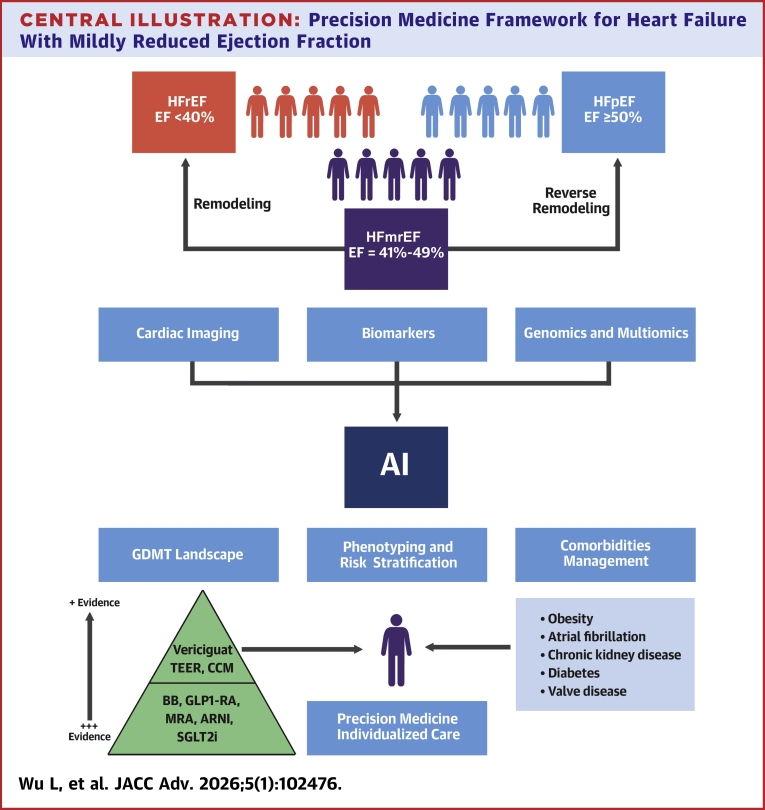
Precision Medicine Framework for Heart Failure With Mildly Reduced Ejection Fraction Schematic representation illustrating the heterogeneity of HFmrEF and the integration of phenotyping, combining advanced imaging (echocardiogram, CMR, and PET), biomarker profiling, and genomic characterization and with artificial intelligence to refine risk stratification, guide individualized therapy, and move beyond ejection fraction–based classification toward data-driven, precision management. AI = artificial intelligence; ARNI = angiotensin receptor–neprilysin inhibitor; BB = beta-blocker; CCM = cardiac contractility modulation; CMR = cardiac magnetic resonance; EF = ejection fraction; GDMT = guideline-directed medical therapy; GLP-1RA = glucagon-like peptide-1 receptor agonist; HFmrEF = heart failure with mildly reduced ejection fraction; HFpEF = heart failure with preserved ejection fraction; HFrEF = heart failure with reduced ejection fraction; MRA = mineralocorticoid receptor antagonist; PET = positron emission tomography; SGLT2i = sodium-glucose cotransporter 2 inhibitor; TEER = transcatheter edge-to-edge repair.

## Conclusions

HFmrEF is a distinct but clinically diverse form of HF, characterized by overlapping features of preserved and reduced EF. Although dedicated clinical evidence remains limited, recent clinical trials have shown promising results, offering a growing range of treatment options for HFmrEF patients. Precision medicine and individualized management using advanced imaging, biomarker profiling, and patient-specific treatment strategies will be essential to optimize outcomes in this under-recognized and clinically heterogeneous population.

## Funding support and author disclosures

The authors have reported that they have no relationships relevant to the contents of this paper to disclose.
